# Low-Molecular-Mass Fragments of Collagen Improve Parameters Related to Mass and Inflammation of the Adipose Tissue in the Obese Rat

**DOI:** 10.17113/ftb.61.01.23.7926

**Published:** 2023-03

**Authors:** Olesia Kalmukova, Nataliia Raksha, Tetiana Vovk, Tetiana Halenova, Mykola Dzerzhynsky, Dinko Mitrecic, Olexiy Savchuk, Ludmila Ostapchenko

**Affiliations:** 1 Department of Biochemistry, Educational and Scientific Centre “Institute of Biology and Medicine”, Taras Shevchenko National University of Kyiv, 64/13, Volodymyrska Street, Kyiv, Ukraine; 2 Department of Cytology, Histology and Reproductive Medicine, Educational and Scientific Centre “Institute of Biology and Medicine”, Taras Shevchenko National University of Kyiv, 64/13, Volodymyrska Street, Kyiv, Ukraine; 3 Department of Histology and Embryology, University of Zagreb School of Medicine, Šalata 3, Zagreb, Croatia

**Keywords:** high-calorie diet, visceral and subcutaneous adipose tissue, fibrosis, crown-like structures, bioactive peptides, mast cells

## Abstract

**Research background:**

Despite clearly recognized links between increased body mass and increased risk for various pathological conditions, therapeutic options to treat obesity are still very limited. The aim of the present study is to explore the effect of low-molecular-mass collagen fragments obtained from the scales of Antarctic wild marine fish on rats' visceral and subcutaneous white adipose tissue in a high-calorie diet-induced obesity model.

**Experimental approach:**

The study was conducted on outbred rats, which were divided into 3 experimental groups: (*i*) control, consuming standard food (3.81 kcal/g), (*ii*) obese group, consuming a high-calorie diet (5.35 kcal/g), and (*iii*) obese group, consuming a high-calorie diet (5.35 kcal/g) with intragastric administration of low-molecular-mass collagen fragments (at a dose 1 g/kg of body mass during 6 weeks). Low-molecular-mass collagen fragments were obtained by a procedure that included collagen extraction from fish scales and enzymatic hydrolysis with pepsin. Apart from hematoxylin and eosin staining, fibrosis level was assessed by histochemical Van Gieson’s trichrome picrofuchsin staining, and mast cells were analysed by toluidine blue O staining.

**Results and conclusions:**

Group treated with low-molecular-mass fragments of collagen exhibited decreased rate of mass gain, relative mass, area occupied by collagen fibre of both visceral and subcutaneous adipose tissue, and cross-sectional area of both visceral and subcutaneous adipocytes. Treatment with low-molecular-mass fragments of collagen reduced the infiltration of immune cells, number of mast cells and their redistribution back to the septa. This was also accompanied by a decreased number of the crown-like structures formed by the immune cells, which are markers of chronic inflammation that accompanies obesity.

**Novelty and scientific contribution:**

This is the first study that reports the anti-obesity effect of low-molecular-mass fragments produced as a result of controlled hydrolysis of collagen from the scales of Antarctic wild marine fish in the *in vivo* model. Another novelty of this work is the observation that the tested collagen fragments not only reduce the body mass, but also improve the morphological and inflammatory parameters (decrease in the number of crown-like structures, immune cell infiltration, fibrosis and mast cells). Altogether, our work suggests that low-molecular-mass collagen fragments are a promising candidate for amelioration of some comorbidities linked to obesity.

## INTRODUCTION

Worldwide, at least 2.8 million people die each year as a result of being overweight or obese, and an estimated 35.8 million (2.3%) of global deaths occur due to comorbidities associated with being overweight or obese (*1*). According to the World Health Organization data, 39% of adults were overweight and 13% were obese in 2016. The intensive development of the COVID-19 pandemic has in addition worsened the prevalence of obesity and accelerated the emergence of complications associated with it (diabetes type 2, hypertension, metabolic syndrome, oncological and cardiovascular diseases, *etc.*) (*2*). Although a lot of efforts have been invested in searching for therapy, not many options are available, since obesity is a complex multifactor disease (*3*).

One of the promising directions in the treatment of various pathological conditions, including obesity, presented in this work is based on bioactive peptides. Bioactive peptides are simple, natural, low-cost molecules derived from plants and animals with many proven beneficial effects (*4*). For example, one study reported free radical scavenging, redox balance and wound healing activity (*5*). In another study, in addition to anti-inflammatory properties, antihypertensive properties were also shown; collagen hydrolysates from tape jellyfish had an inhibitory effect on the angiotensin-converting enzyme (*6*). In cultured human dermal fibroblasts, collagen-derived bioactive peptides increased elastin synthesis, while significantly inhibiting the release of MMP-1 and MMP-3 and elastin degradation (*7* ). Bioactive peptides obtained as a result of enzymatic hydrolysis of the collagen of turkey by-products were able to bind both cholic and deoxycholic acids and inhibit plasma amino oxidase; therefore, they had a hypocholesterolaemic effect (*8*). In experiments based on atherosclerosis mice model, various bioactive peptides in the collagen hydrolysate from *Salmo salar* skin protected endothelial cell injury and regulated inflammation, oxidative stress as well as platelet aggregation (*9*). Marine collagen peptides in type 2 diabetic patients decreased significantly the amounts of free fatty acids, resistin, prostacyclin cytochrome P450, leptin and nitric oxide, while amounts of adiponectin and bradykinin rose markedly (*10*). In male C57Bl6/J mice after diet-induced obesity, fish collagen peptides slowed down the increase in body mass, fat mass, basal glycaemia, and the amount of inflammatory cytokines (*11*).

The most interesting category of peptides that attracted a lot of attention are collagen bioactive peptides derived from animal by-products (*12*), including those derived from organisms that live in extreme conditions (*13*). They include molecules derived from cold-adaptive hydrobionts. They differ from mammalian collagen in some physicochemical properties and amino acid composition (*14*). Although there are some publications reporting on the use of skin and scales of freshwater and marine fish for the extraction of type I collagen (*15*), there is no data on the effects of collagen fragments obtained from the scales of the fish living in the Antarctic region.

Although sources of collagen are available and often quite cheap, frequent outbreaks of infectious diseases in terrestrial animals necessitate the search for alternative sources of proteins and peptides. In addition, the use of animal-derived molecules in everyday life may be inappropriate for some patients due to their religious beliefs. In this regard, hydrobionts can be considered a potential source of bioactive molecules, including peptides. Currently, bioactive peptides of marine origin are of considerable interest, and, due to a wide range of bioactivity and high absorption efficiency, are considered as potential agents for the pharmaceutical and nutraceutical industries.

In our previous studies the low-molecular-mass collagen (LMMC) fragments derived from the scales of the Antarctic wild marine fish showed promising effects in diet-induced obesity model, namely: lowering of blood glucose, glycated haemoglobin and serum insulin concentrations. We hypothesized that the observed effects might be used to reduce mass and ameliorate obesity-associated comorbidities (*16*). Thus the main goal of our study is to analyze the effect of LMMC on visceral and subcutaneous white adipose tissue in high-calorie diet-induced obese rats.

## MATERIALS AND METHODS

### Preparation of low-molecular-mass collagen fragments

**Fig. 1:**
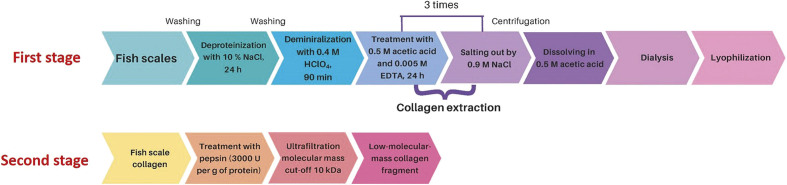
General scheme of two-stage preparation of low-molecular-mass collagen fragments below 10 kDa derived from Antarctic fish scales by enzymatic hydrolysis with pepsin under controlled conditions

To obtain low-molecular-mass collagen (LMMC) fragments, scales of the mackerel icefish (*Champsocephalus gunnari, Nototheniidae*) from the Antarctic region were used. Briefly, wild marine fish were caught by the Ukrainian Antarctic expeditions near the Galindez island (geographical coordinates: 65°15’S, 64°15’W). The scales were washed thoroughly with distilled water and stored until use. Collagen was extracted from fish scales in two steps (*17*) with modifications (Fig. 1).

First, the scales were immersed in 10% NaCl (at a ratio of *m*(dry scale)/*V*(solution)=1:10) to remove proteins. Then, the fish scales were demineralized with a 0.4 M HClO_4_ (a ratio of *m*(dry scale)/*V*(solution)=1:15) for 90 min and washed three times with distilled water. To extract collagen, demineralized scales were mixed with 0.5 M acetic acid containing 0.005 M EDTA and stirred for 24 h. The sample was centrifuged (Allegra TM64R; Beckman Coulter, Indianapolis, IN, USA) at 10 000×*g* for 30 min. The obtained precipitate was dissolved in 0.5 M acetic acid containing 0.005 M EDTA and stirred for 24 h. After centrifugation (10 000×*g* for 30 min), collagen was extracted by adding NaCl (at a final concentration of 0.9 M) to the supernatant. The step of collagen extraction was repeated twice. The obtained collagen was dialyzed against distilled water and lyophilized.

The fraction of LMMC fragments was produced as described previously (*18*). The lyophilized collagen (1 g) was dissolved in 20 mL of 0.2 M acetic acid and mixed with pepsin (~2500 U per mg protein; Sigma-Aldrich, Merck, St. Louis, MO, USA) at the ratio of *m*(enzyme)*m*(collagen)=1:100. The mixture was stirred at 37 °C for 8 h. To stop the hydrolysis, the sample was heated at 95 °C for 10 min in a temperature-controlled water bath shaker. The sample was centrifuged at 10 000×*g* for 30 min. Collagen fragments with a molecular mass of less than 10 kDa were separated by ultrafiltration using a Pierce™ Protein Concentrator PES, 10K MWCO (Thermo Fisher Scientific, Waltham, MA, USA). The obtained fraction of low-molecular-mass collagen fragments was lyophilized with laboratory freeze-dryer LyoQuest-55 (Telstar, Bensalem, PA, USA).

Sodium dodecyl sulfate polyacrylamide gel electrophoresis (SDS-PAGE) at 8 and 15% was used to check the purity of the obtained collagen and to estimate the molecular mass of the collagen fragments, respectively. SDS-PAGE was carried out according to the method of Laemmli (*19*). Lyophilized collagen or collagen peptides were dissolved in sample buffer (0.05 M Tris, pH=8.8, 2% SDS, 5% sucrose and 0.02% bromophenol blue) to a concentration of 1 mg/mL. The samples were heated at 95 °C for 1 min before loading into polyacrylamide gel. The total amount of proteins or collagen fragments loaded per lane of the gel was 20 µg. For negative control sample, citrate buffer was used, since the lyophilized collagen fragments were dissolved in 0.05 M citrate buffer (pH=5.0)

The electrophoresis was performed using the Mini-Protean Tetra System (Bio-Rad Laboratories, Inc, Hercules, CA, USA) at 19 mA for stacking and 36 mA for separating gels. The gels were stained with 2.5% Coomassie brilliant blue R-250 and (in *φ*/%): ethanol 10, acetic acid 10 and isopropanol 15. To calculate the molecular mass of collagen or collagen peptides, the Natural High-Range SDS-PAGE Standards and Natural Polypeptide SDS-PAGE Standards (Bio-Rad Laboratories, Inc) were used. The electropherograms were analyzed using the TotalLab v. 2.04 program (*20*).

### High-calorie diet-induced obesity model and study groups

Outbred male Wistar rats (total of 60 animals) aged 2 months, initial mass (110±10) g, were used for the experiment. Experimental design was accepted by the Ethical Committee of ESC ’Institute of Biology and Medicine’ Taras Shevchenko National University of Kyiv, Ukraine. In addition, this study was made in agreement with the Dir 2010/63/EU of the European Parliament (*21*), and the Article 26 of the Law of Ukraine On the Protection of Animals from brutal treatment (*22*).

After acclimation during 7 days, the rats were randomly separated into 2 groups: (*i*) control group (20 animals) received standard food Purina rodent chow (3.81 kcal/g; VІТА, Obukhiv, Ukraine) and water, and (*ii*) obesity-modeling group (40 animals) received high-calorie diet (HCD: 5.35 kcal/g) that contained 60% standardized food Purina rodent chow (VІТА), 10% pork visceral fat, 10% chicken eggs, 9% sugar from beets, 5% raw peanuts, 5% powdered whole milk (26% fat), 1% vegetable (sunflower) oil and water (*23*). When the average body gain between groups demonstrated significant difference by at least 30%, the second group (40 animals) was divided into 2 equal groups (20 animals per group): without treatment (HCD group) and with daily administration of LMMC (HCD+LMMC group). LMMC was administered by gavage tube at a dose of 1 g/kg of body mass, dissolved in 0.05 M citrate buffer (pH=5.0), daily for 6 weeks.

In the animal facility standard conditions of temperature ((22±3) °C), humidity ((60±5) %), and light (12:12 h) were constantly provided. Аll rats had *ad libitum* access to food and water. Consumption of food and water were measured daily from 9 until 10 a.m. (relative daily food (kcal per day per g of body mass) and relative daily water consumption (mL per day per rat) were determined). Rats were weighed weekly (body mass gain (%) and rate of mass gain (%) were calculated for each rat).

After the euthanasia by guillotine, white visceral (the epididymal, retroperitoneal and perirenal parts) and subcutaneous (inguinal part) adipose tissue were weighed for the relative visceral and subcutaneous fat mass determination.

### Histology analysis

For histology analysis of the visceral white adipose tissue (WAT) the perirenal part was used, and for the analyses of the subcutaneous WAT, the inguinal part was used. Tissue was fixed in 4% neutral buffered paraformaldehyde (Sigma-Aldrich, Merck) for 72 h. From the tissue embedded in formalin-fixed paraffin 5 µm thick sections were obtained and stained with hematoxylin (Merck, Darmstadt, Germany) and eosin (HLR, Kyiv, Ukraine).

The histological assessment of the inflammation was carried out according to the generally accepted semi-quantitative scoring scale for the presence of immune infiltration: absence 0, mild 1, moderate 2 and strong 3.

### Fibrosis detection in WAT

To analyze the amount of tissue fibrosis, Van Gieson's histochemical trichrome method was used to detect collagen fibre (*24*). Histological slides were stained with picrofucsin (100 mL saturated aqueous picric acid (Sfera Sim, Lviv, Ukraine) mixed with 10 mL of 1% aqueous acid fuchsin solution (HLR, Kyiv, Ukraine) and hematoxylin to visualize nuclei (*24*). The quantitative measuring of red collagen fibre (the related area occupied by collagen fibre) was determined as a percentage of the total tissue area.

### Mast cell content in WAT

Histochemical staining with a 0.1% aqueous solution of toluidine blue O (Carl Roth GmbH, Karlsruhe, Germany) was used to detect the mast cells (*25*). All slides were observed by a light microscope Olympus BX41 (Tokyo, Japan) with Olympus DP20 (Tokyo, Japan) digital camera and the QuickPHOTO MICRO software (Promicra, Prague, Czech Republic) (*26*). Morphometric parameters were calculated using ImageJ software (*27*).

### Statistical analysis

For all morphometric analyses we used five slides of one staining method from each experimental animal. Kolmogorov-Smirnov test was used for the determination of data distribution normality. One-way ANOVA with Tukey’s *post hoc* multiple comparison tests served for the assessment of significance of the observed changes, while in the case of nonparametric method, Kruskal–Wallis test for independent samples was used. A statistically significant difference was evaluated at p<0.05 using Statistica v. 7.0 (*28*). The obtained results are presented as mean value±standard error of the mean (SEM).

## RESULTS AND DISCUSSION

**Fig. 2:**
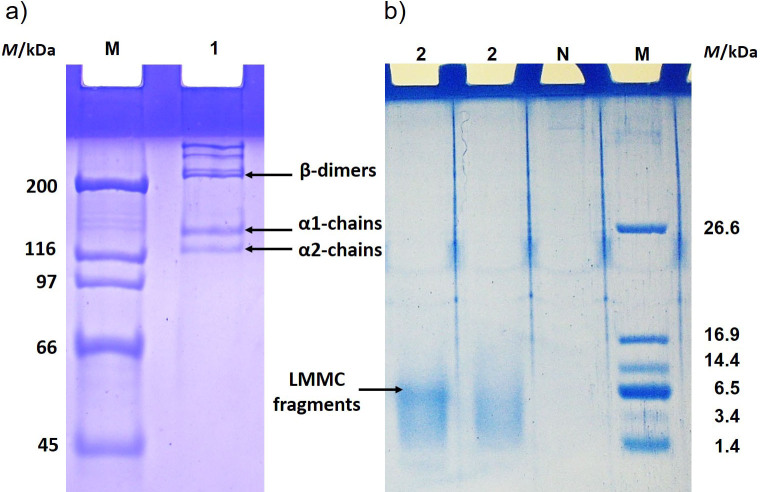
Electropherogram of collagen extracted from fish scales: a) collagen visualized before digestion by using 8% polyacrylamide gel (lane 1), M=molecular mass markers, b) low molecular mass collagen fragments obtained by enzymatic digestion visualized by using 15% polyacrylamide gel, lane 2=collagen fragments (LMMC). N=negative control sample, *M*=molecular mass, LMMC=low-molecular-mass collagen

To confirm the purity of collagen isolated from fish scales, SDS-PAGE in 8% polyacrylamide gel was performed. According to the results (Fig. 2a), the extracted collagen belongs to type I collagen, as it consists of α1-chains (about 127-130 kDa) and α2-chains (about 116-120 kDa). As can be seen from Fig. 2a, the intensity of α1-chains is stronger than that of α2-chains, hence the amount of α1-chains was greater than α2-chains. This may be an additional confirmation that the collagen from the fish scales of the Antarctic region belongs to type 1 collagen. Proteins with a molecular mass above 200 kDa are β1- and β2-dimers, which are the result of the dimerization of α-chains.

To obtain low-molecular-mass collagen fragments, a two-stage method was used; enzymatic hydrolysis of fish scale collagen with pepsin and subsequent ultrafiltration of a mixture of fragments of different molecular mass using 10 kDa molecular mass cut-off (MMCO) membranes. This approach made it possible to obtain the fraction of collagen fragments with a molecular mass below 10 kDa (Fig. 2b), which were used to study their effect on the development of high-calorie diet-induced obesity.

### General changes in body mass parameters

In our model, HCD caused a significant increase in the general parameters of body mass: mass gain of the HCD animals was on average increased by 39%, which was a significant difference compared to control. Although the application of LMMC ameliorated the increase in body mass gain, no statistically significant difference was observed in comparison with the control group. In comparison with the HCD group, the LMMC-treated group demonstrated a 12% lower increase in the mass gain (Table 1).

**Table 1:** General body mass changing parameters, relative daily water and food consumption of experimental groups

Parameter	Experimental group		
Control	HCD	HCD+LMMC
Body mass gain/%	195±23	(271±17)*	238±23
Rate of last week mass gain/%	26.4±1.7	(37.3±3.3)*	(27.6±1.9)^#^
Relative mass of visceral fat/%	1.0±0.2	(2.9±0.3)*	(1.78±0.03)*^#^
Relative mass of subcutaneous fat/%	1.43±0.08	(1.9±0.2)*	(1.34±0.08)^#^
Relative food consumption per body mass/(kcal/(day·g))	0.39±0.03	(0.59±0.06)*	0.48±0.05
Relative water consumption per rat/ (mL/day)	38.9±0.7	(32.2±0.4)*	(30.8±0.4)*

Data are presented as the mean value±SEM; HCD=high-calorie diet, HCD+LMMC=high-calorie diet group receiving low-molecular-mass collagen. *р<0.05 compared with control value, ^#^р<0.05 compared with HCD

While the rate of mass gain in the HCD group was significantly increased, by 41%, compared to the control group, the same parameter in HCD+LMMC group increased by only 4% (not significant increase). Thus, the difference between the treated (LMMC) and untreated (HCD) group was 26% and this difference was statistically significant.

The relative visceral fat mass in our model was three times higher in HCD than in the control group. Treatment with LMMC caused statistically significant decrease in relative visceral fat mass, by 40%, compared to HCD, but still this amount differed significantly from the control group.

The relative mass of the subcutaneous fat in the HCD group significantly increased by 31% compared to the control group. The same parameter after LMMC treatment was significantly reduced, by 29%, compared to the HCD group, reaching amounts even below those in the control group (Table 1).

Relative daily food consumption in the group treated with LMMC was also decreased, although this difference did not reach statistical significance. Interestingly, when relative daily water consumption was analyzed, both HCD and HCD+LMMC groups showed significantly decreased levels compared to the control group, by 17 and 20% respectively. We believe that this might be caused by a high-calorie diet, which contains more water than a standard diet.

There are several studies which tested different additives with a goal to reduce obesity and body mass gain. Thus for example, black soybean (*29*) was given to diet-induced obese mice and soy protein β-conglycinin (*30*) to male Sprague-Dawley rats. In these experiments, general body mass parameters were changed, but different to our experiment with LMMC, those changes were obtained by general reduction of appetite. Some studies, again based on soy peptides, like for example the one based on BALB/c mice and a soy peptide aglycin, did not observe differences in body mass parameters or food intake compared to the diabetic model control (*31*). Another study based on soy hydrolysates reported a reduction in fat pad mass (in this case also decreased body gain in genetically obese KK mice) without general (body composition) effects on body mass and food consumption (*32*). When animal source of biogenic peptides for potential anti-obesity effect are taken in account, similar data have been reported after tests with egg-derived hydrolysates/peptides and Wistar rats: lower value of food intake, body and fat mass in adipose and non-adipose tissues (*33*). Other experiments reported reduced fat accumulation in non- adipose tissues without changes of body mass and food intake (*34*). Another interesting report was based on the casein glycomacropeptide hydrolysates. It affected high-fat diet and streptozotocin-induced diabetic mice by modulating gut microbiota and caused significantly decreased overall body mass (*35*). Significant mass losses were also noticed after administration of pyroglutamyl leucine (PyroGlu-Leu) to mice with dextran sulfate sodium-induced colitis (*36*) and of rapeseed peptides to mice with d-galactose induced ageing (*37*).

### Morphological analyses of the white adipose tissue

**Fig. 3:**
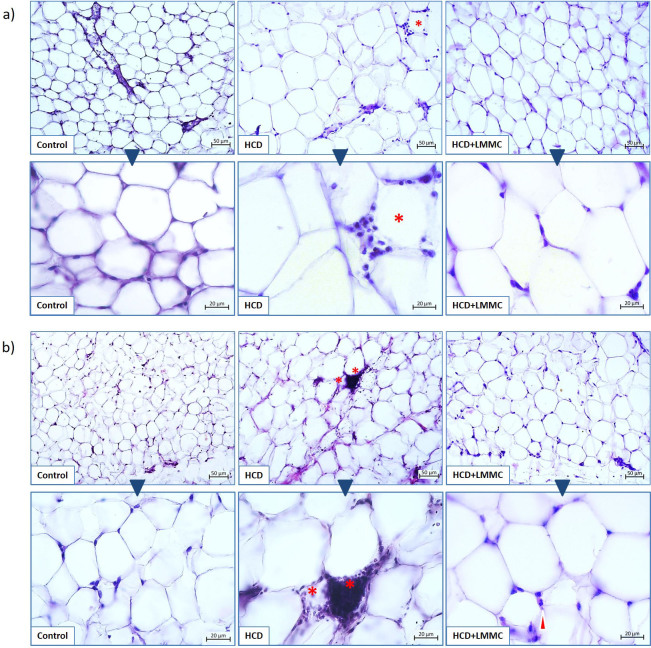
Microphotographs of: a) rats’ visceral and b) subcutaneous white adipose tissue of experimental groups: control, HCD and HCD+LMMC. Hematoxylin and eosin staining. Asterisk=crown-like structure, arrow head=granulocyte infiltration. HCD=high-calorie diet, LMMC=low-molecular-mass collagen

Histological evaluation of the visceral and subcutaneous white adipose tissue (WAT) was performed using the hematoxylin/eosin stain (Fig. 3).

Dominant finding when visceral WAT was analyzed after 6 weeks of the treatment included hyperplasia and hypertrophy of adipocytes with marked infiltration of the immune cells, which suggests the presence of the low-grade tissue inflammation (Fig. 3a). Immune cells around adipocytes initiated enhanced apoptotic mechanisms that cause the appearance of crown-like structure (CLS), a prominent feature of chronic adipose-tissue inflammation and indication of dying adipocytes (*38*). Application of LMMC caused a significant decrease in the number of immune cells infiltrated in the visceral WAT. While infiltration of immune cells in HCD group was 7.6 times higher than in the control group (HCD (2.3±0.2) points, control group (0.3±0.2) points), in LMMC+HCD group this parameter was twice lower than in the HCD group (LMMC+HCD (1.2±0.2) points).

Analyses of the subcutaneous WAT of the HCD group showed an increased presence of immune cells (macrophages and lymphocytes), appearance of CLS and a thickened and rough extracellular matrix which surrounds each adipocyte (Fig. 3b).

The administration of LMMC resulted in the reduction of infiltration of immune cells in subcutaneous WAT and of thickness of the extracellular matrix, which was shown as a smoother border between adipocytes. While the infiltration of immune cells in the subcutaneous WAT of obese rats was 4 times (significantly) higher than in the control group, the administration of LMMC resulted in a significant decrease (50%) of infiltration than in the HCD group (control group (0.4±0.2), HCD (1.6±0.2), LMMC+HCD (0.8±0.2) points). Another interesting finding came from the observation of the presence of granulocytes. While in the control group this type of cells is regularly present in healthy subcutaneous WAT, they are much rarer in HCD group. Interestingly, after the treatment with LMMC, their presence in the subcutaneous WAT was reestablished (arrow head in Fig. 3). On the other hand, in visceral WAT granulocytes are not so commonly found. This finding is in agreement with some published reports (*39*).

**Fig. 4:**
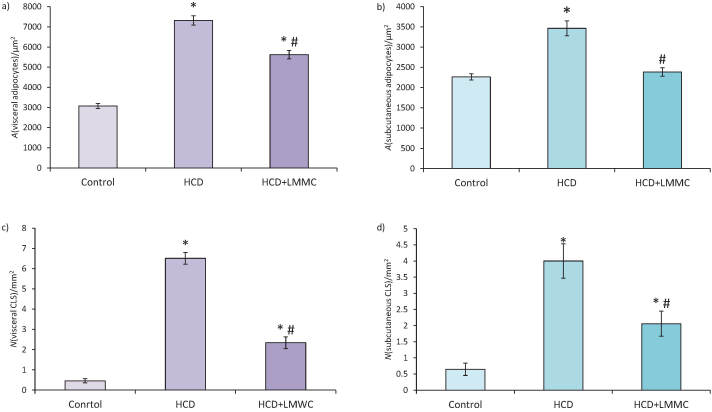
Results of morphometric analysis: cross-section area (*A*) of: a) visceral and b) subcutaneous white adipocytes, and the number (*N*) of c) visceral and d) subcutaneous crown-like structures (CLS). Data are presented as the mean value±SEM; *р<0.05 compared with control value, р<0.05 compared with HCD. HCD=high-calorie diet, LMMC=low-molecular-mass collagen

Another important parameter that we analyzed was the cross-sectional area of adipocytes (Fig. 4).

In our HCD model, the cross-sectional area of visceral WAT adipocytes (Fig. 4a) increased 2.4 times, which was a statistically significant finding compared to the control group. Treatment of animals with LMMC caused a 23% decrease of the cross-sectional area of visceral WAT adipocytes, which was recognized as statistically significant compared to HCD group. However, in HCD+LMMC group, the area of visceral WAT adipocytes significantly increased, by 83%, compared to the control group.

In HCD rats the cross-sectional area of subcutaneous WAT adipocytes (Fig. 4b) also significantly increased, by 53%, compared to the control. The administration of LMMC decreased the cross-sectional area of subcutaneous WAT by 31% compared to the HCD group, *i.e*. it reached the control value. Thus, LMMC significantly decreased cross-sectional area of both subcutaneous and visceral WAT adipocytes, although this effect was stronger in the subcutaneous WAT, where it reached control levels. This can be explained by more dramatic hypertrophic changes in visceral than in subcutaneous WAT during obesity. These results indicated that the LMMC attenuated adipocyte growth and the accumulation of adipose tissue when consuming high-calorie diet.

Another prominent parameter in the analyses of low-grade inflammation during development of obesity was the number of crown-like structures (CLS).

In our HCD model, the number of CLS in visceral WAT (Fig. 4c) significantly increased, 14 times *vs* control. The treatment of animals with LMMC caused a 2.8 times decrease in the number of CLS in visceral WAT compared to HCD group, which was recognized as statistically significant. Still, this parameter was 5 times higher than in the control group.

The number of CLS in subcutaneous WAT (Fig. 4d) of obese rats was 6 times higher than in the control. In the treated animals, this parameter decreased twice compared to the HCD group, which was a statistically significant finding. Still, this was 3 times higher than in the control group.

In the HCD group, the number of CLS increased more noticeably in the visceral than in subcutaneous WAT. LMMC treatment resulted in partial improvement in CLS number as it had intermediate values between HCD and control group in visceral and subcutaneous WAT both.

Our analyses of WAT changes in obesity model after the use of bioactive peptides can be compared to some other reports. Thus, the application of collagen peptides from *Walleye pollock* skin in a high-fat diet-fed mice led to reduced size of adipocytes, epididymal and subcutaneous fat mass and lower body mass gain without food intake changes *via* diminished serum levels of triglycerides (*40*). On the other hand, a study based on the egg white hydrolysate administration to Zucker obese rats reported decreased adipose tissue mass and lipid accumulation without changes in morphological parameters (*41*). A study based on administration of bioactive peptides from plants, capsanthin and capsaicin to obesity-induced C57BL/6J mice reported a reduction of adipocyte size in inguinal and epididymal fat pad and decrease of inguinal and epididymal fat pad mass only after an intake of a high dose of capsanthin-enriched extract pellets (*42*). In addition, there are some synthetic peptides that have recently been tested as anti-obesity molecules. For example, a novel liposome-encapsulated peptide PDBSN reduced the body mass, subcutaneous, visceral and epididymal fat mass and significantly diminished adipocyte volume in high-fat diet-induced obese mice (*43*).

There are some *in vitro* studies that also reported the activity of bioactive peptides on adipocytes. Experiments performed on 3T3-L1 embryonic mouse cells investigated some effects on the obesity-related parameters, such as up-regulated (soluble soy protein peptic hydrolysate (*44*)) or down-regulated expression of peroxisome proliferator-activated receptor (novel anti-obesity peptide (RLLPH) derived from hazelnut (*Corylus heterophylla* Fisch) protein hydrolysates (*45*)), down-regulated gene expression of lipoprotein lipase and fatty acid synthase (protein hydrolysates from β-conglycinin-enriched soybean genotypes (*46*)), inhibited CCAAT-enhancer-binding proteins (or C/EBPs) and adipocyte differentiation (Asp-Ile-Val-Asp-Lys-Ile-Glu-Ile peptide from tuna (*47*)), decreased inflammatory responses and insulin resistance in adipocytes (Phe–Leu–Val peptide from soy (*48*)), and significantly decreased accumulation of triglycerides (peptides isolated from *Spirulina platensis* proteins (*49*)). In primary rat preadipocytes culture, casein glycomacropeptide from cheese whey inhibited cell proliferation and differentiation (*50*).

### Analyses of fibrosis and the number of mast cells in WAT

We hypothesized that chronic low-grade inflammation in HCD-induced obesity rat model influenced the extracellular matrix. Thus we performed analyses of the level of tissue fibrosis and the presence of mast cells in WAT with the goal to detect possible positive influence of LMMC on these parameters.

**Fig. 5:**
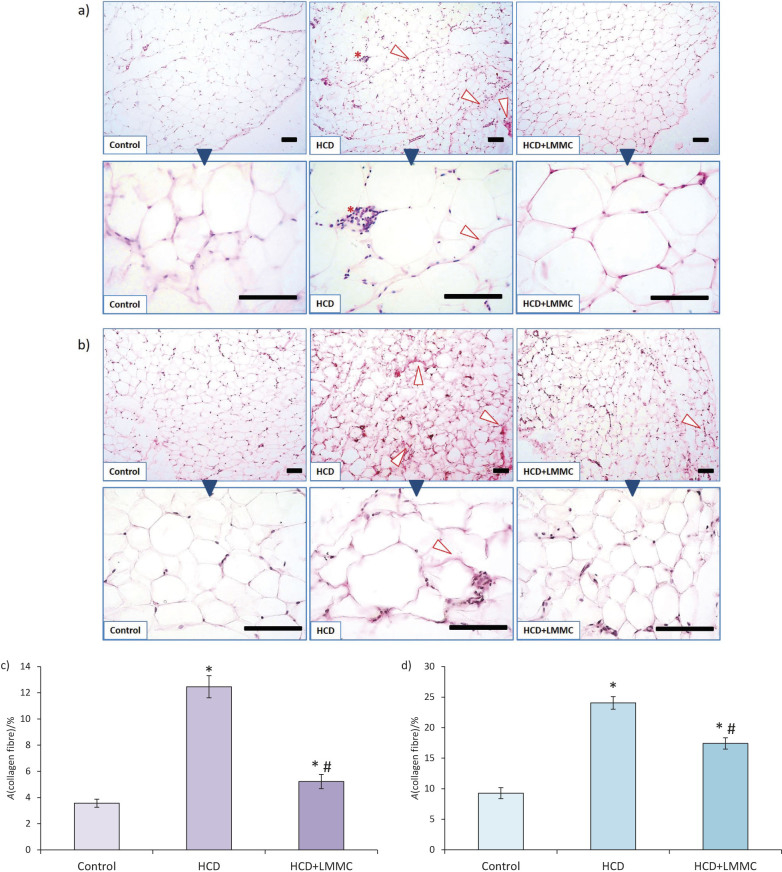
Microphotographs of the rat: a) visceral and b) subcutaneous white adipose tissue of experimental groups (control, high-calorie diet (HCD) and HCD with low-molecular-mass collagen (HCD+LMMC) group). Van Gieson’s picrofuchsin histochemical staining of extracellular matrix; scale bar 100 μm. Asterisk=crown-like structure, arrow head=massive collagen fibre. Results of fibrosis amounts (relative area (*A*) occupied by collagen fibre) in: c) visceral and d) subcutaneous white adipose tissue (WAT). Data are presented as the mean value±SEM, *р<0.05 compared with control value, ^#^р<0.05 compared with HCD

In visceral WAT of the control group (Fig. 5a), large collagen fibre was present only in the connective tissue capsule.

As opposed to this, in the obese rats (HCD group) we found huge collagen fibre near CLS (asterisk in Fig. 5), around hypertrophic adipocytes forming a trap of collagen bundles and between lobules in connective septa (arrow head in Fig. 5). The administration of LMMC prevented the growth of massive collagen fibre in all three above-mentioned places (Fig. 5c). In our HCD model, the relative area occupied by collagen fibre in visceral WAT was 3.5 times higher than in the control value, which was statistically significant. Treatment with LMMC caused the area occupied by collagen fibre to decrease by 58%, which was recognized as statistically significant. At the same time, the relative area occupied by the collagen fibre in HCD+LMMC group was still significantly higher, by 47% than in the control group.

Changes in obese rats were more emphasised in the subcutaneous (Fig. 5b) than in visceral WAT. The quantity of the extracellular matrix increased, which affected the shape of enlarged adipocytes too. Thus, they became surrounded by rigid fibre, making the borders between cells to appear rough. The difference in the observed manifestation of fibrosis between visceral and subcutaneous WAT can be at least partly explained by the difference in the physiological role of these fat pads. In addition to the role in energy metabolism and endocrine secretion, subcutaneous WAT performs biomechanical functions, like absorption of trauma and body insulation (*51*). Massive collagen deposits in the HCD group were also noticed in the regions of connective septa, walls of blood capillaries and at the sites of CLS. The administration of LMMC attenuated obesity-caused changes; the borders between adipocytes regained more smooth appearance with reduced amount of rigid fibre. The same improvement in the morphology was observed in septa and capillaries. In the HCD group, relative area occupied by collagen fibre in subcutaneous WAT was 2.6 times higher than in the control animals (Fig. 5d). LMMC administration significantly decreased the relative area occupied by the collagen fibre in subcutaneous WAT, by 28%, although this parameter was still increased by 88% compared to the control group. Thus, the administration of LMMC significantly decreased the level of fibrosis in both subcutaneous and visceral WAT, although this parameter was still significantly higher than in the control group.

**Fig. 6:**
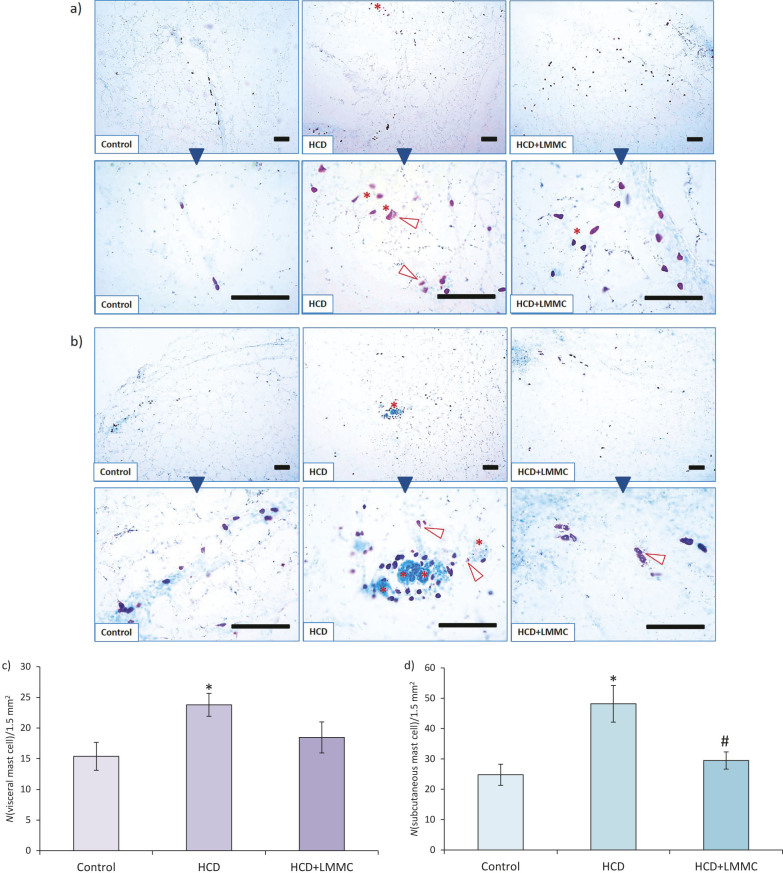
Microphotographs of rat: a) visceral and b) subcutaneous white adipose tissue (WAT) of experimental groups (control, high-calorie diet (HCD) and HCD with low-molecular-mass collagen (HCD+LMMC) group), histochemical staining for mast cell detection (purple). Toluidine blue staining; scale bar 100 μm. Asterisk=crown-like structure, arrow head=degranulated mast cells. Results of the analysis of the presence of mast cells: in c) visceral and d) subcutaneous WAT. Data are presented as the mean value±SEM, *р<0.05 compared with control value, ^#^р<0.05 compared with HCD

Adipose tissue infiltration by immune cells in obese rats included activation of mast cells that were involved in fibrosis development. In lean control, visceral adipose tissue mast cells were located around blood capillaries and some in the septa (Fig. 6a).

When we analysed the location of mast cells in the visceral WAT, we noticed that in the HCD group they migrated from the connective septa inside the lobules, which also resulted in their massive presence in the CLS (asterisk in Fig. 6). In addition, we observed that in the HCD group most of the mast cells were in a degranulated state. This indicated their strong impact on the development of a low-grade chronic inflammation. The administration of LMMC decreased the number of mast cells and caused their relocation into their typical places, near capillaries and septa, as in the control group. Moreover, the LMMC caused most of the mast cells to return to their inactive, non-degranulated state. In subcutaneous WAT, mast cells demonstrate similar changes as in visceral WAT, in accordance with the features of different groups (Fig. 6b).

Analyses of the number of mast cells revealed the following: in subcutaneous WAT of the HCD group, the number of mast cells increased twice compared to the control (Fig. 6d). The administration of LMMC decreased their number by 39%, which was a statistically significant reduction that reached a level not different from the control group. On the other hand, such strong effect was not found in the visceral WAT (Fig. 6c). In visceral WAT of the HCD group, the number of mast cells increased by 55% *vs* control. The administration of LMMC decreased the number of mast cells, but this reduction did not reach statistical significance.

In this study we report the data that suggest a decrease of the inflammation in WAT after the application of LMMC, *i.e.* a decreased appearance of CLS and level of fibrosis, alongside with decreased number of mast cells that are in non-degranulated (not activated) state both in visceral and subcutaneous WAT. These observed elements at least partly explain how the application of LMMC fragments can lead to a decrease in the rate of mass gain and relative visceral fat mass.

These data are in congruence with our results of cytokines analysis, in which we reported that the application of LMMC in obesity model reduced the level of pro-inflammatory cytokines IL-1β and IL-12 and increased the level of anti-inflammatory cytokines IL-4 and IL-10, and partly restored superoxide dismutase and catalase activities in the serum (*16*). Similar results are reported in other studies which applied bioactive peptides: milk casein-derived tripeptide Val-Pro-Pro reduced macrophage infiltration into the adipose tissue (phenotype M1 macrophages (F4/80^+^/CD11c^+^ cells)) and suppressed inflammatory gene expression (MCP-1, a monocyte or macrophage chemotactic factor) in high-fat diet-induced murine adipose tissue inflammation (*52*). In a similar model, the attenuated expression of the proinflammatory cytokines TNF-α and IL-1β in WAT *via* an angiotensin‐converting enzyme (ACE)-dependent cascade was reported (*53*). Other reported mechanisms of promotion of adipocyte differentiation and inhibited inflammation were shown *in vitro* on 3T3-F442A cells through the suppression NF-κB pathway by milk-derived tripeptides IPP (Ile-Pro-Pro) and VPP (Val-Pro-Pro) (*54*). Another interesting study was based on bitter melon (*Momordica charantia*); its extracts attenuated obesity-associated macrophage and mast cell infiltration, and pro-inflammatory cytokine expression in adipose tissues of C57BL/6 mice with high-fat diet (*55*).

At present, considerable interest is directed to the study of possible mechanisms through which bioactive peptides achieve positive effects. This includes anti-inflammatory effects (inhibition of the NF-κB, MAPK and JAK-STAT pathways), antioxidant effects (decreased reactive oxygen species and cyclooxygenase activity), modulation of gut microbiome, cholesterol-lowering effects (connected to bile acids/salts or inhibition of cholesterol solubility, affecting cholesterol biosynthesis, increase of LDL uptake and cholesterol degradation), regulation of expression of the nuclear transcription factor PPAR, action on glycaemic parameters and insulin signalling (inhibitors of α-amylase, α-glucosidase, sodium glucose co-transporter-2 inhibitors, plasma-based dipeptidyl peptidase-4 (DPP4) or insulin mimetics and production of GLP-1) and antihypertensive effects (suppression of angiotensin I-converting enzyme) (*56*).

## CONCLUSIONS

This is the first study which reports that low-molecular-mass fragments of collagen originating from the scales of Antarctic wild marine fish administered at a dose per body mass of 1 g/kg during six weeks show a clear anti-obesity effect. The complex two-stage method applied in the work for obtaining collagen fragments under controlled conditions makes it possible to produce a mixture of peptides with a molecular mass of less than 10 kDa. They not only reduce the body mass, but also improve morphological and inflammatory parameters: a decrease in hypertrophy of adipocytes and number of crown-like structures, *i.e.* markers of chronic inflammation, alongside with the infiltration of immune cells and number of mast cells were observed both in visceral and subcutaneous white adipose tissue of diet-induced obese rats. Equally, low-molecular-mass fragments reduced the measured parameters of fibrosis. Altogether, our work suggests that low-molecular-mass collagen fragments represent a promising candidate for amelioration of comorbidities linked to obesity.
